# Differential impact of neutrophil-to-lymphocyte ratio and time-weighted NLR on mortality and survival in critically ill children: insights from a retrospective study

**DOI:** 10.3389/fped.2025.1559405

**Published:** 2025-05-20

**Authors:** Dongqing Ma, Wei Wang, June Ma, Wei Liu

**Affiliations:** ^1^ Department of Pediatrics, Civil Aviation General Hospital, Beijing, China; ^2^ Department of Surgical Intensive Care Unit, Beijing Anzhen Hospital, Capital Medical University, Beijing, China

**Keywords:** neutrophil-to-lymphocyte ratio, critically ill children, mortality, survival, timeweighted NLR

## Abstract

**Background:**

The intensive care of critically ill children is challenging due to diverse etiologies and rapid disease progression. Early identification of high-risk patients is crucial for improving outcomes. The neutrophil-to-lymphocyte ratio (NLR) has emerged as a potential biomarker reflecting the balance between innate and adaptive immune responses, with studies in adults showing its correlation with mortality and survival in intensive care settings. However, its application in pediatric intensive care units (PICU) is less explored.

**Objective:**

To examine the impact and predictive value of NLR on in-hospital mortality and 90-day survival rates in critically ill children using data from the Pediatric Intensive Care (PIC) database at the Children's Hospital of Zhejiang University School of Medicine.

**Methods:**

This retrospective cohort study included 3,350 patients from the PIC database, with patients older than 28 days and an ICU stay of at least 48 h. Data on demographic information, ICU admission type, laboratory test results, and clinical outcomes were collected. The normal range of NLR was calculated using the percentile method. Time-weighted NLR was calculated using the trapezoidal rule to estimate the area under the curve of NLR values over time. Statistical analyses included chi-square tests, Mann–Whitney *U* tests, and multivariable logistic regression to assess the association between NLR and outcomes.

**Results:**

Higher time-weighted NLR levels were significantly associated with increased in-hospital mortality (OR = 1.15, 95% CI: 1.08–1.22, *p* < 0.001) and shorter ICU length of stay. The Kaplan–Meier survival analysis showed significantly different 90-day survival rates among NLR groups (*p* = 0.034). Subgroup analysis revealed the highest predictive value of time-weighted NLR in patients under 1 year old with an initial NLR less than 0.48 (AUC = 0.832).

**Conclusion:**

The study confirms NLR, particularly in its time-weighted form, as a robust prognostic indicator for critically ill children. Elevated time-weighted NLR levels are associated with increased in-hospital mortality and shorter ICU stays, highlighting its potential for risk stratification and guiding clinical decisions in PICU. The dynamic nature of the time-weighted NLR provides a more accurate reflection of the patient's inflammatory burden over time. However, the retrospective and single-center design of the study limits the generalizability of the results. Future research should address these limitations and explore the integration of NLR with other clinical and laboratory parameters to enhance prognostic accuracy in pediatric critical care settings.

## 1 Introduction

The intensive care of critically ill children presents unique challenges due to the diverse etiologies of their conditions and the rapid progression of disease that can lead to severe outcomes. Mortality and survival rates are critical metrics for evaluating the effectiveness of care provided in pediatric intensive care units (PICU). Early identification of patients at high risk for adverse outcomes is essential for guiding clinical interventions and improving patient outcomes. However, predicting mortality and survival in critically ill children remains challenging, as it involves a complex interplay of various clinical, laboratory, and demographic factors.

The Neutrophil-to-Lymphocyte Ratio (NLR) has emerged as a potential biomarker in recent years, reflecting the balance between innate and adaptive immune responses. Neutrophils are the first line of defense against infections, while lymphocytes orchestrate antigen-specific adaptive immunity (1). The ratio of these two cell types can provide insights into the systemic inflammatory response and stress levels in critically ill patients. Several studies have explored the predictive value of NLR in various clinical settings, including sepsis, cardiovascular diseases, and cancer, demonstrating its potential as a prognostic indicator (2–4).

In adult populations, NLR has been shown to correlate with mortality and survival outcomes in intensive care settings. For instance, a study by Salciccioli et al. (2) found that NLR was significantly associated with mortality in critically ill patients. Similarly, Wu et al. (3) reported that NLR could predict mortality in intensive care unit patients, highlighting its potential as a low-cost and easily accessible biomarker. However, the application of NLR in pediatric populations, particularly in the context of PICU, is less explored. The few existing studies have provided mixed results, emphasizing the need for further investigation (5, 6).

Given the differences in immune responses and disease presentations between adults and children, it is crucial to evaluate the predictive value of NLR specifically in pediatric intensive care settings. This study aims to bridge this gap by examining the impact and predictive value of NLR on in-hospital mortality and 90-day survival rates in critically ill children. By leveraging data from the Pediatric Intensive Care (PIC) database at the Children's Hospital of Zhejiang University School of Medicine, we seek to provide insights into the role of NLR as a prognostic indicator in this vulnerable patient population.

## 2 Materials and methods

### 2.1 Study design and setting

This retrospective cohort study was conducted using data from the Pediatric Intensive Care (PIC) database at the Children's Hospital, Zhejiang University School of Medicine. The study aimed to evaluate the impact of the NLR on in-hospital mortality, 28-day mortality (defined as death occurring within 28 days of admission) and **90-day survival rates** (tracked from admission to 90 days post-discharge), and Intensive Care Unit (ICU) Length of Stay (LOS) in critically ill children.

### 2.2 Data source and patient selection

The Pediatric Intensive Care (PIC) database is a large, single-center, pediatric-specific database that contains de-identified clinical data of patients admitted to critical care units at the Children's Hospital of Zhejiang University School of Medicine (7). The initial screening included 13,941 ICU stay records.

The selection criteria were as follows:

• Inclusion Criteria: Patients older than 28 days, patients with an ICU stay at least 48 h.
• Exclusion Criteria: Records with insufficient or missing critical information, Neutrophil, or lymphocyte values of less than 2, patients with concurrent cancer, extreme outliers.

Patients with extreme outliers in the initial NLR were excluded to avoid skewed distributions. Outliers were defined as values beyond 1.5 × IQR from the 25th or 75th percentiles, calculated as:

Lower bound = Q1−1.5 × IQR, Upper bound = Q3 + 1.5 × IQR

Given NLR is a positive ratio, the effective lower bound was set to 0. This method follows CLSI C28-A3 guidelines for reference intervals (8).

**Figure 1:**
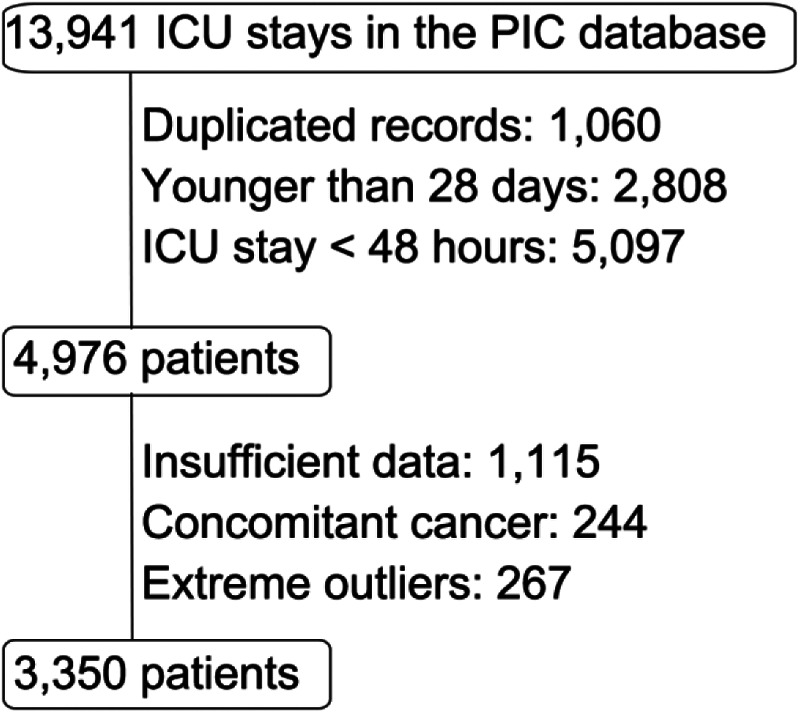
Flow chart of patient selection from the Pediatric Intensive Care (PIC) database.

The selection process is illustrated in the flowchart (Figure 1). The final study population consisted of 3,350 patients.

Data was extracted from the PIC database and included demographic information, type of ICU admission, laboratory test results, and clinical outcomes. Specific laboratory parameters collected were white blood cell count, neutrophil count, lymphocyte count, platelet count, C-reactive protein (CRP), hemoglobin, creatinine, pH, arterial partial pressure of oxygen (PaO2), lactate, potassium, and sodium.

### 2.3 NLR normal range calculation

To ascertain the normal range of the NLR in children over 28 days of age, a rigorous methodology was employed. Initially, data were meticulously selected from the PIC database, ensuring that only records with both neutrophil and lymphocyte absolute values deemed normal were included. Subsequently, outliers in these values were identified and exercised using the Interquartile Range (IQR) technique. 32,860 samples were finally retained. The NLR was then calculated for the cleansed dataset, and its normal range was established by determining the 2.5th (p2.5) and 97.5th (p97.5) percentiles, employing the percentile method—a conventional approach in medical research for defining biomarker reference intervals, aligns with CLSI C28-A3 guidelines for reference intervals, a methodology widely adopted in pediatric biomarker studies (8). This methodical process culminated in the identification of a reliable NLR normal range for the specified pediatric cohort (0.48–6.58), offering a substantive foundation for clinical assessments and investigative pursuits.

### 2.4 Statistical analysis

Continuous variables were assessed for normality using skewness and kurtosis tests and were summarized as mean ± standard deviation if normally distributed, or as median (interquartile range) otherwise. Categorical variables were compared using the chi-square test, while continuous variables were analyzed with the Mann–Whitney *U* test for non-normal distributions and the *t*-test for normal distributions.

The time-weighted NLR calculation method used in this study involves several key steps. Initially, the NLR is calculated at each time point by dividing the neutrophil count by the lymphocyte count. Subsequently, the time-weighted value is computed using the trapezoidal rule, which estimates the Area Under the Receiver Operating Characteristic Curve (AUC) of NLR values over time. This approach averages the NLR values at consecutive time points and multiplies by the time interval between them. Finally, the total AUC is normalized by dividing it by the total duration of the observation period, yielding the time-weighted NLR.

The chi-square test was utilized to evaluate the association between time-weighted NLR groups and binary outcomes such as hospital and 28-day mortality. The Kruskal–Wallis's rank sum test was applied to assess differences in the distribution of ICU LOS across the three NLR groups. The AUC was utilized to assess the discriminative power of the time-weighted NLR in predicting in-hospital mortality within each subgroup.

A multivariable logistic regression analysis was conducted to assess the adjusted odds ratios (OR) for in-hospital mortality among patients. The model was adjusted for time-weighted NLR, initial NLR, pH, Lactate, and PaO2. The mean Variance Inflation Factor (VIF) was calculated to check for multicollinearity. The stepwise selection process was applied with a significance level of *p* < 0.05, and variables that were non-significant in the Wald test were removed from the final model.

Longitudinal NLR trends were analyzed using linear mixed-effects models with random intercepts for individual patients, incorporating time, group (survivors vs. non-survivors), and their interaction term. All analyses were performed using Stata 17.0 and R 4.4.1.A *p*-value of less than 0.05 was considered statistically significant.

## 3 Results

### 3.1 Baseline characteristics and laboratory values

The study included 3,350 patients, categorized into survivors (*n* = 3,112) and non-survivors (*n* = 238) (Table 1). There were no significant differences between the two groups in terms of age and gender. However, significant differences were observed in the type of ICU admitted to, with a higher proportion of non-survivors admitted to the General ICU (*p* < 0.001). Non-survivors exhibited higher levels of C-reactive protein (CRP) (*p* = 0.014), creatinine (*p* < 0.001), and lactate (*p* < 0.001), and lower pH (*p* < 0.001) and arterial partial pressure of oxygen (PaO2) (*p* < 0.001) compared to survivors. Additionally, non-survivors had a higher initial NLR (*p* = 0.041) and time-weighted NLR (*p* = 0.008), a greater proportion of positive blood cultures (*p* = 0.007), and longer ICU stays (*p* < 0.001).

**Table 1:** Baseline characteristics and laboratory values of patients.

Variables	Survivors	Non-survivors	*P*
	(*n* = 3,112)	(*n* = 238)	
Age (years)	0.91 (0.28–3.07)	0.74 (0.24–2.23)	0.100
Gender [*n* (%)]			0.143
Male	1,744 (56.0%)	145 (60.9%)	
Female	1,368 (44.0%)	93 (39.1%)	
Type of ICU [*n* (%)]			
CICU	791 (25.4%)	15 (6.3%)	<0.001
SICU	324 (10.4%)	19 (8.0%)	0.234
PICU	1,107 (35.6%)	89 (37.4%)	0.572
NICU	251 (8.1%)	16 (6.7%)	0.461
General ICU	639 (20.5%)	99 (41.6%)	<0.001
Laboratory test			
Lymphocyte (10^9^/L)	2.3 (1.5–3.5)	2.5 (1.5–4.3)	0.111
Neutrophil (10^9^/L)	5.1 (3.0–8.1)	4.6 (2.4–8.4)	0.163
Platelet (10^9^/L)	236 (148–348)	269 (140–389)	0.229
CRP (mg/L)	9 (2.2–40)	5 (0.7–25)	0.014
Hemoglobin (g/L)	105.2 ± 20.9	104.5 ± 22.8	0.646
Creatinine (umol/L)	38 (29–47)	42.5 (31–56)	<0.001
PH	7.38 ± 0.09	7.32 ± 0.16	<0.001
PaO_2_ (mmHg)	132 (70–179)	80 (51–145)	<0.001
Lactate (mmol/L)	1.8 (1.2–2.7)	2.1 (1.4–3.9)	<0.001
Potassium (mmol/L)	3.7 (3.4–4.2)	3.9 (3.4–4.3)	0.001
Sodium (mmol/L)	137 (134–140)	137 (133–141)	0.874
Initial NLR	2.16 (1.15–3.82)	1.96 (0.82–4.80)	0.041
Time-weighted NLR	2.72 (1.33–4.74)	3.20 (1.59–5.35)	0.008
PLR	97.4 (59.5–155.9)	101.9 (52.6–161.0)	0.835
Positive blood culture	270 (8.7%)	33 (13.9%)	0.007
Sepsis	120 (3.9%)	8 (3.4%)	0.701
ICU LOS (day)	6.0 (3.8–12.1)	10.0 (4.6–22.0)	<0.001

NLR, neutrophil-to-lymphocyte ratio; PLR, platelet-to-lymphocyte ratio; PaO_2_, arterial partial pressure of oxygen; CRP, C-reactive protein; LOS, length of stay.

### 3.2 Impact of NLR on mortality and ICU length of stay

Patients were categorized into three groups based on their time-weighted NLR values: <0.48, 0.48–6.58, and >6.58 (Table 2). The in-hospital mortality rate increased with higher time-weighted NLR levels, with rates of 4.1%, 6.8%, and 10.0% for NLR < 0.48, 0.48–6.58, and >6.58, respectively, showing a statistically significant difference (*p* = 0.024). The 28-day mortality rate also showed an increasing trend, but without statistical significance (*p* = 0.062). The median ICU LOS was significantly lower for the group with NLR > 6.58 at 4.7 days compared to 7.0 days for NLR < 0.48 and 6.6 days for NLR 0.48–6.58 (*p* = 0.0001).

**Table 2:** Unadjusted results of time-weighted NLR for patients.

Outcome	Time-weighted NLR			*P*
	<0.48 (*n* = 145)	0.48–6.58 (*n* = 2,783)	>6.58 (*n* = 52)	
In-hospital mortality, *n* (%)	6 (4.1%)	190 (6.8%)	42 (10.0%)	0.024
28-day mortality, *n* (%)	5 (3.5%)	156 (5.6%)	34 (8.1%)	0.062
ICU LOS (day), median (IQR)	7.0 (3.9–12.9)	6.6 (3.9–13.6)	4.7 (2.9–8.1)	0.001

NLR, neutrophil-to-lymphocyte ratio; LOS, length of stay.

Mixed-effects modelling revealed a significant interaction between time and survival status (*β* = 0.036, *p* < 0.001), indicating divergent NLR trajectories between survivors and non-survivors (Supplementary Table S1, Supplementary Figure S1).

### 3.3 Multivariable logistic regression analysis

A multivariable logistic regression analysis was conducted to assess the adjusted odds ratios (OR) for in-hospital mortality among patients. The final model included time-weighted NLR, pH, lactate, PaO2, and initial NLR. The results showed that time-weighted NLR (OR = 1.15, 95% CI: 1.08–1.22, *p* < 0.001), pH (OR = 0.08, 95% CI: 0.02–0.28, *p* < 0.001), lactate (OR = 1.09, 95% CI: 1.03–1.41, *p* < 0.001), and PaO2 (OR = 0.99, 95% CI: 0.991–0.995, *p* < 0.001) were significantly associated with in-hospital mortality. initial NLR was inversely associated with in-hospital mortality (OR = 0.90, 95% CI: 0.84–0.97, *p* = 0.005) (Table 3).

**Table 3:** Adjusted in-hospital mortality risk of multivariable analysis.

Variable	Risk of mortality adjusted OR	95% CI	*p*
Time weighted NLR	1.15	1.08–1.22	<0.001
Initial NLR	0.90	0.84–0.97	0.005
PH	0.08	0.02–0.28	<0.001
Lactate	1.09	1.03–1.41	<0.001
PaO_2_	0.99	0.991–0.995	<0.001

NLR, neutrophil-to-lymphocyte ratio; VIF, variance inflation factor; The mean VIF was 4.4.

### 3.4 Kaplan–Meier survival analysis

**Figure 2:**
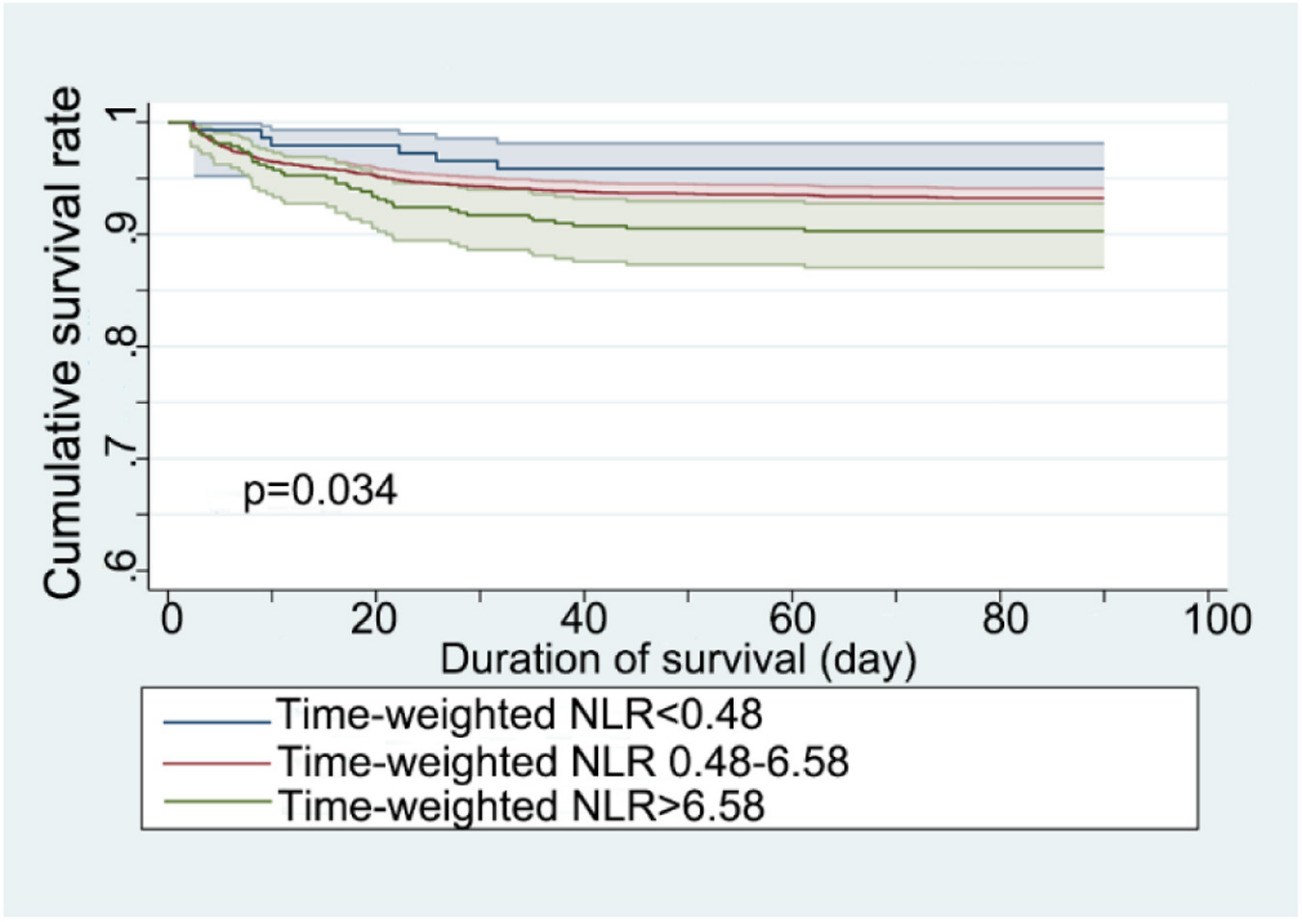
Kaplan–Meier survival curve for 90-day mortality stratified by time-weighted NLR (neutrophil-to-lymphocyte ratio).

The Kaplan–Meier survival curve assessed the 90-day survival rates of patients stratified by time-weighted NLR levels (Figure 2). The survival curves were significantly different among the three NLR groups, with a *p*-value of 0.034 from the Log-rank test.

### 3.5 Receiver operating characteristic (ROC) curve analysis

**Figure 3:**
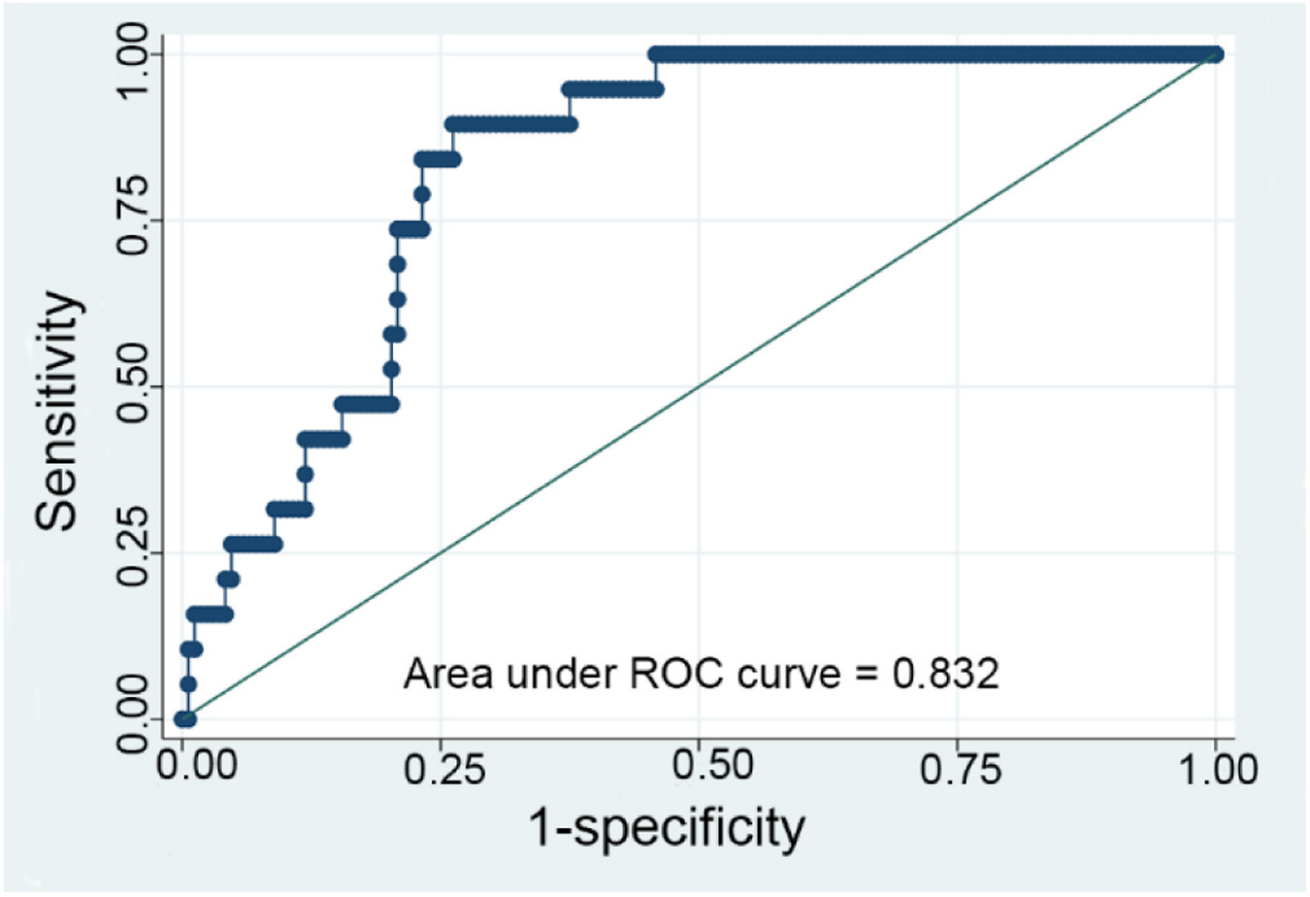
ROC curve for the time-weighted NLR (neutrophil-to-lymphocyte ratio) model with subgroup analysis analyzing a subgroup of patients with an initial NLR less than 0.48 and aged less than 1 year.

The time-weighted NLR demonstrated varying predictive values across different subgroups (Table 4). Notably, the subgroup with the highest predictive value was patients less than 1 year old with an initial NLR less than 0.48, yielding an AUC of 0.832 (Figure 3). This indicates a strong discriminative ability of the time-weighted NLR in this specific subgroup. In contrast, the other subgroups exhibited lower predictive values.

**Table 4:** Predictive performance of time-weighted NLR for in-hospital mortality across different subgroups.

Subgroup	All patients	Patients age <1	Patients age ≥1
Time-weighted NLR	AUC = 0.552	AUC = 0.563	AUC = 0.577
<0.48	AUC = 0.285	NA	AUC = 0.359
0.48–6.58	AUC = 0.528	AUC = 0.536	AUC = 0.567
>6.58	AUC = 0.452	AUC = 0.405	AUC = 0.460
Initial NLR	AUC = 0.460	AUC = 0.493	AUC = 0.443
<0.48	AUC = 0.684	AUC = 0.832	AUC = 0.459
0.48–6.58	AUC = 0.565	AUC = 0.548	AUC = 0.620
>6.58	AUC = 0.564	AUC = 0.511	AUC = 0.602

NLR, neutrophil-to-lymphocyte ratio; AUC, area under the receiver operating characteristic curve.

## 4 Discussion

The present study provides valuable insights into the role of the NLR as a prognostic marker in critically ill children, particularly in predicting in-hospital mortality and ICU LOS. The findings demonstrate that higher time-weighted NLR levels are significantly associated with increased in-hospital mortality and shorter ICU LOS, highlighting the potential utility of NLR in risk stratification and clinical decision-making in PICU.

### 4.1 Correlation of NLR with in-hospital mortality: insights from multivariable analysis

The neutrophil-lymphocyte ratio (NLR) has been extensively studied as a potential prognostic marker for in-hospital mortality across various clinical conditions. In our study, multivariable logistic regression analysis reveals a positive correlation between time-weighted NLR and in-hospital mortality, suggesting that elevated NLR values over time are associated with increased mortality risk (OR = 1.15, 95% CI: 1.08–1.22, *p* < 0.001). Conversely, initial NLR shows a negative correlation with mortality (OR = 0.90, 95% CI: 0.84–0.97, *p* = 0.005), indicating that higher initial NLR values may be associated with a decreased risk of mortality, which is counterintuitive and requires further investigation. To further elucidate the paradoxical inverse association between initial NLR and mortality, we performed a longitudinal analysis using a linear mixed-effects model. This model accounted for repeated NLR measurements within individual patients and revealed distinct temporal NLR trajectories between survivors and non-survivors. Survivors exhibited a significant decline in NLR over time (slope = −0.039/day, *p* < 0.001), indicative of resolving inflammation. In contrast, non-survivors showed minimal NLR reduction (slope = −0.003/day), suggesting persistent inflammatory dysregulation (time × group interaction: *β* = 0.036, *t* = 7.31, *p* < 0.001). These findings underscore that single-timepoint NLR measurements (e.g., initial NLR) may fail to capture critical dynamic immune responses. Specifically, survivors' transiently elevated initial NLR could reflect early stress-induced lymphopenia, whereas non-survivors' lower initial NLR might signal impaired neutrophil mobilization during later-stage immune exhaustion. The time-weighted NLR, by integrating these temporal dynamics, provides a more robust prognostic marker than static measurements. In specific clinical settings, such as acute heart failure (AHF) (9), ST-elevation myocardial infarction (STEMI) (10), and acute respiratory distress syndrome (ARDS) (4), NLR has been established as an independent risk factor for in-hospital mortality, with early increases in NLR linked to higher mortality rates. In contrast, in conditions like sepsis (11), and in acute decompensation of alcohol-related liver cirrhosis (ALC) (12), the relationship is less straightforward, with higher NLR values correlating with lower mortality risk, contradicting prior research. In large middle cerebral artery (MCA) infarction patients (13), NLR shows a weak positive correlation with length of hospital stay but does not independently predict in-hospital mortality. These findings underscore the complexity of NLR as a prognostic marker, highlighting its utility in some conditions while suggesting limitations in others. Further research is necessary to elucidate the mechanisms underlying these associations and to refine the clinical application of NLR as a prognostic tool.

In adults, NLR has been extensively validated in CAP (community-acquired pneumonia) cohorts. For instance, de Jager et al. (14) demonstrated that NLR predicts severity and mortality in CAP (AUC = 0.701), while Cataudella et al. (15) highlighted its prognostic value in elderly CAP patients. Although our study lacked CAP-specific subgroup analyses due to heterogeneous PICU etiologies, these adult studies underscore NLR's potential utility in pediatric pneumonia, warranting future targeted investigations. Unlike adults, where baseline NLR reflects neutrophilic hyperactivation (16), pediatric NLR is confounded by developmental immune immaturity. As highlighted by Buonacera et al. (1), delayed neutrophil mobilization and stress-induced lymphopenia decouple baseline NLR from outcomes, necessitating dynamic monitoring. Our findings align with this framework, demonstrating that time-weighted NLR—not single-timepoint values—provides actionable prognostic insights in children. Recent work by Regolo et al. (17) in adult COVID-19 patients demonstrated that NLR independently predicts mortality (HR = 1.77), while CRP correlates more strongly with ICU admission (HR = 1.70). Their mediation analysis further revealed that CRP's impact on PaO_2_/FiO_2_ ratio (P/F) is partially mediated by neutrophils, aligning with our observation of divergent NLR/CRP trajectories in survivors vs. non-survivors. Although pediatric immune dynamics differ (e.g., delayed neutrophilia), these findings emphasize the need to explore NLR-CRP-P/F interplay in future pediatric studies, particularly in hypoxemic respiratory failure.

### 4.2 Mechanisms underlying NLR dysregulation

The NLR serves as a comprehensive indicator of systemic inflammation (18), reflecting the balance between neutrophils (19), which are key players in the innate immune response, and lymphocytes, which are essential for adaptive immunity (20). An elevated NLR indicates a dysregulation of this balance, with an overactive innate immune response and a suppressed adaptive immune response. This can exacerbate the severity and progression of critical illnesses, leading to tissue damage, organ dysfunction, and increased mortality rates (3, 4, 9, 11). The overactivation of neutrophils can trigger a cytokine storm, amplifying the inflammatory response and creating a vicious cycle of inflammation (21). Lymphopenia, often indicated by a high NLR, may result from stress-induced apoptosis (22), sequestration in inflamed tissues (23), or impaired production in the bone marrow (24). Additionally, inflammation-induced endothelial dysfunction can increase vascular permeability and micro thrombosis, impairing organ perfusion and leading to multi-organ failure (25). Clinically, an elevated NLR at admission has been shown to predict worse outcomes, including longer hospital stays, higher complication rates, and increased mortality (26). Monitoring changes in NLR over time can help clinicians assess treatment responses and adjust therapeutic strategies accordingly (27).

### 4.3 Pediatric vs. adult immune responses: implications for NLR interpretation

The divergent prognostic utility of NLR between pediatric and adult critically ill populations—highlighted by the superior predictive value of time-weighted NLR over baseline NLR in children—likely stems from fundamental differences in immune system maturation and inflammatory dynamics. As highlighted by Buonacera et al. (1), NLR in adults reflects neutrophilic hyperactivation (e.g., rapid cytokine release in sepsis or COVID-19), whereas pediatric NLR dynamics are governed by: (1) developmental immaturity in neutrophil reserves and bone marrow mobilization, leading to delayed neutrophilia and transiently suppressed NLR during early critical illness (28); (2) blunted inflammatory kinetics, particularly in infants, where single-timepoint NLR fails to capture evolving immune dysregulation, necessitating dynamic monitoring via time-weighted NLR to quantify cumulative inflammatory burden (29, 30); and (3) age-dependent lymphocyte dynamics, characterized by naïve T-cell predominance and impaired regulatory T-cell function, which predispose children to rapid lymphopenia under stress, further decoupling baseline NLR from outcomes (31, 32). Additionally, developmental variances in endothelial-coagulation interactions, may weaken NLR's association with organ failure compared to adults (33, 34). These mechanisms collectively underscore why time-weighted NLR, rather than static NLR, emerge as a pediatric-specific prognostic tool. Future studies should establish age-stratified NLR thresholds and integrate functional immunophenotyping (e.g., neutrophil CD64, lymphocyte subsets) to optimize risk stratification in PICU.

### 4.4 Subgroup analysis and clinical implications

The analysis of the time-weighted NLR in this study provides significant insights into its predictive value for in-hospital mortality across various patient subgroups. The highest AUC value of 0.832 was observed in patients under 1 year old with an initial NLR less than 0.48, indicating a strong predictive ability in this specific cohort. Lower AUC values in other subgroups suggest that the predictive power of the time-weighted NLR may be influenced by patient age and the initial inflammatory state. This highlights the importance of considering patient age and initial inflammatory status when interpreting NLR values, as these factors can significantly affect the prognostic accuracy of the time-weighted NLR.

The shorter ICU LOS in patients with elevated time-weighted NLR appears to reflect dual mechanisms: in survivors, high NLR may signify an intense but transient inflammatory response followed by rapid recovery, enabling earlier discharge. In non-survivors, high NLR is associated with prolonged LOS due to progressive organ failure. Competing risk analysis confirmed that elevated NLR independently predicts mortality (HR = 1.05) (Supplementary Table S8), but its relationship with LOS is contingent on survival status. These findings underscore the importance of dynamic NLR monitoring rather than relying on single-timepoint measurements.

The combined model incorporating NLR, CRP, and lactate demonstrated significantly higher discriminative ability (AUC = 0.609) compared to time-weighted NLR alone (AUC = 0.552) (Supplementary Table S9), suggesting that integrating inflammatory and metabolic markers better reflect the multifactorial nature of critical illness. However, the modest AUC highlights the need for future studies to incorporate dynamic immune-functional parameters (e.g., lymphocyte subsets or neutrophil CD64) to optimize prognostic accuracy.

The findings from the subgroup analysis have important clinical implications. The time-weighted NLR can be a valuable tool for risk stratification, particularly in very young patients, helping to identify those at high risk of mortality early in their hospital course. This can facilitate more aggressive interventions and closer monitoring. However, the variability in NLR predictive power across different subgroups suggests that a one-size-fits-all approach may not be optimal. Future studies should explore age-specific and inflammation-state-specific models to enhance the prognostic accuracy of NLR. Additionally, combining NLR with other biomarkers or clinical parameters may further improve its predictive capacity. Understanding these factors can help clinicians better utilize NLR in clinical decision-making and improve patient outcomes.

### 4.5 Limitations and future research directions

Despite the promising findings, this study has limitations requiring acknowledgment. The retrospective single-center design introduces selection bias and limits generalizability, particularly given the exclusion of 1,115 cases with missing NLR data—a subgroup exhibiting higher mortality that may have skewed the cohort toward less severe inflammatory profiles. Although sensitivity analyses demonstrated robustness (Supplementary Table S3), unmeasured immune dysregulation in excluded cases remains concerning. Methodological constraints further include: (1) missing FiO₂ data precluding PaO₂/FiO₂ ratio analysis; (2) incomplete anthropometric parameters preventing eGFR calculation; and (3) unaccounted confounders such as genetic predispositions and comorbidities, combined with a lack of pathogen-specific data (e.g., viral vs. bacterial infection types), which may critically influence NLR dynamics and subgroup interpretations—particularly in infants where viral infections (e.g., RSV) may disproportionately affect lymphocyte counts. Notably, while adult studies associate NLR/CRP with CAP severity (14, 15) and neutrophil-mediated respiratory failure (17), our dataset lacked standardized CAP classifications and P/F measurements, hindering pediatric-adult mechanistic comparisons. Future multicenter studies should prioritize standardized biomarker collection, incorporate genetic and comorbidity profiling, and validate NLR's predictive utility across diverse pediatric populations through integrated clinical-laboratory parameter analyses.

## 5 Conclusion

This study substantiates the NLR, particularly in its time-weighted form, as a robust prognostic indicator for critically ill children. Elevated time-weighted NLR levels were found to be significantly associated with increased in-hospital mortality and shorter ICU stays, underscoring its potential for risk stratification and guiding clinical decisions in PICU. The dynamic nature of the time-weighted NLR provides a more accurate reflection of the patient's inflammatory burden over time, offering a distinct advantage over static measures. While our findings are promising, the study's retrospective and single-center design limits the generalizability of the results. Future research should address these limitations and further explore the integration of NLR with other clinical and laboratory parameters to enhance prognostic accuracy and improve patient outcomes in pediatric critical care settings.

## Data Availability

Publicly available datasets were analyzed in this study. This data can be found here: PIC Database http://pic.nbscn.org.
