# Supplementary material for: Differential impact of neutrophil-to-lymphocyte ratio and time-weighted NLR on mortality and survival in critically ill children: insights from a retrospective study
Source: Front Pediatr. 2025 May 20;13:1559405. doi: 10.3389/fped.2025.1559405 (PMC12130025; doi:10.3389/fped.2025.1559405)
Supplement: Supplementary file 1 [file Datasheet1.pdf]

Supplementary

Table S1. Longitudinal mixed-effects model results for NLR trajectories.

Predictor	Estimate	SE	t-value	P
Intercept	2.705	0.026	103.211	<0.001
time_day	-0.039	0.002	-22.665	<0.001
group	-0.049	0.098	-0.499	0.618
time_day:group	0.036	0.005	7.306	<0.001

Table S2. Baseline characteristics of excluded vs. included cases.

	Excluded (n=1115)	Included (n=3350)	P
Age (years)	2.77 (0.56-7.20)	0.89 (0.28-3.04)	<0.001
In-hospital mortality, n (%)	179 (16.1%)	238 (7.1%)	<0.001
Gender (n (%))			0.313
Male	648 (58.1%)	1889 (56.4%)	
Female	467 (41.9%)	1461 (43.6%)	
Sepsis	37 (3.3%)	128 (3.8%)	0.441
ICU LOS (day)	6.0 (3.8-12.1)	10.0 (4.6-22.0)	<0.001

Abbreviations: LOS, length of stay.

Table S3. IPW-adjusted logistic regression analysis.

Covariate	Odds Ratio	95% Conf. Interval	p
Time-weighted NLR	1.10	1.04-1.16	<0.001
Age	0.96	0.93-1.01	0.142
ICU LOS	1.01	1.00-1.18	0.003
Gender	1.28	0.97-1.69	0.080
Sepsis	1.01	0.48-2.15	0.976
_cons	0.04	0.03-0.06	<0.001

Abbreviations: _cons, estimates baseline odds; NLR = neutrophil-to-lymphocyte Ratio; IPW = Inverse Probability Weighting; LOS, length of stay.

Figure S1. Dynamic NLR trends in survivors vs. non-survivors.

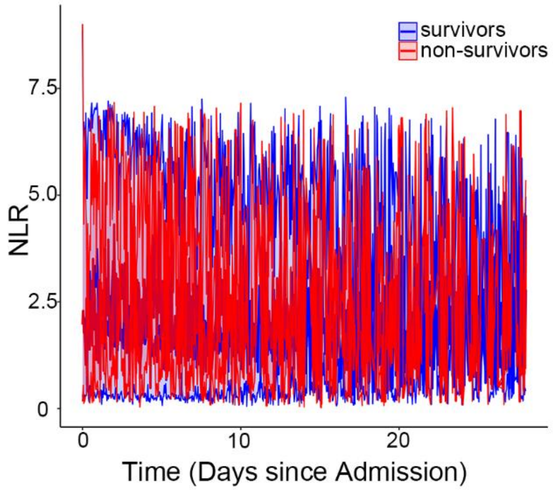

Figure Legend S1: Mixed-effects modelling revealed a significant interaction between time and survival status ($\beta = 0.036$, $p < 0.001$), indicating divergent NLR trajectories between survivors and non-survivors
